# The failure of medical education to develop moral reasoning in medical students

**DOI:** 10.5116/ijme.547c.e2d1

**Published:** 2014-12-27

**Authors:** Vicki S. Murrell

**Affiliations:** Department of Counseling, Educational Psychology and Research, College of Education, Health and Human Sciences, University of Memphis, Tennessee, USA

**Keywords:** Medical education, moral development, medical training, moral development in medical education

## Abstract

**Objectives:**

The goal of this study was to determine differences in moral judgment among students in medical school.

**Methods:**

This cross-sectional study involved students currently enrolled in undergraduate medical education. Recruited via email, 192 students took an online version of the Defining Issues Test to determine their current stage of moral judgment, as well as their percentage of post conventional thought. Independent variables included year of graduation, which indicated curriculum completion as well as participation in a professionalism course. Data was analyzed primarily using One-Way Analysis of Variance.

**Results:**

Of the 192 participants, 165 responses were utilized. ANOVA showed no significant differences in moral judgment between or among any of the student cohorts, which were grouped by year of matriculation. Comparisons included students in the four years of medical school, divided by graduation year; students about to graduate (n=30) vs. those still in school (n=135); and students who had participated in a course in professionalism (n=91) vs. those who had not (n=74).

**Conclusions:**

These results demonstrate a lack of evolution in the moral reasoning of medical students and raise the issue of what might stimulate positive changes in moral judgment during the medical school experience.

## Introduction

 In many parts of the world, both the general public and healthcare educators express great concern about patient safety, quality healthcare, and healthcare effectiveness. At the center lies the discussion about choices made by medical doctors. The training of a physician consists of many complex and multi-dimensional components, but none may be more personal than the process of making ethically complex decisions which, research shows, can be influenced by physician’s personal characteristics, 1, 2personal social values, 3 and moral judgment. 4, 5

 Limited research has been done regarding moral development during medical school. Several research studies have used the theories of cognitive moral development in higher education. 6, 7 However, given the moral imperative that accompanies the work of physicians, 8 the dearth of research regarding moral judgment in medical students is surprising. The research that does exist shows that while increased focus on ethics in the medical school curriculum can yield significant gains in measured moral development, there is a persistent failure of medical education, worldwide, to make the changes to impact the moral judgment of its students. 

###  Theoretical framework: Cognitive moral development 

 Jean Piaget’s theory of cognitive moral development launched deliberations to connect how humans cultivate an individual sense of “right”. 7,9, 10 His theoretical framework included a dialectic wherein an individual’s schema (or way of knowing) would change in order to adapt to unfamiliar stimuli. Ground breaking work by Lawrence Kohlberg, 11 followed by the research of other theorists such as James Rest, 9 Eliot Turiel 12 and Albert Bandura, 13 provided a model of moral reasoning influenced by both cognition and society. Kohlberg’s theory, predominant during the past fifty years, provided the framework for this research. 

 Kohlberg, utilizing Piaget’s epistemology research, postulated that people differentiate between personal needs and societal conventions. He theorized that interactions with society and situations provided a necessary dialectical antagonist for moral development. 14 Kohlberg believed that when met with situations that opposed or threatened their current moral judgment, individuals would adjust their perspectives in order to make sense of their experiences. This integration of newfound knowledge would promote the growth of the individual’s moral reasoning and produce a qualitative change in the individual’s thinking processes. 14,15

 Kohlberg’s theory consists of three levels, each representing a fundamental shift in the individual’s moral perspective-taking. Each level contains two stages. Kohlberg maintained that movement through the stages and levels was invariant, sequential, cross-cultural, and universal. The preconventional level reflects an egocentric perspective. Within the next level, the conventional level, the focus shifts from the self to what is generally accepted by a specific group or society (thus the use of the term “conventional”). The post conventional level reflects thinking that is beyond both the self and the rules and regulations of society. 

### Review of the literature

 A general assumption is that medical education is responsible for helping future doctors develop the ability to exercise ethical judgment in the face of immense responsibility, unpredictability, and constant advances in the practice of medicine. 16 Undergraduate medical education (i.e., the four years of medical school) consists of a complex curriculum involving didactic courses in the basic sciences (generally during the first two years, referred to as M1 and M2) and active learning under the supervision of preceptors during the clinical years (M3 and M4). In 1985, the Liaison Committee on Medical Education (LCME), the accrediting body of medical colleges in North America, created a standard requiring medical colleges to teach those ethical, behavioral, and socioeconomic topics pertinent to the practice of medicine. 17 There has never been any standardization of curricula to satisfy those teaching objectives, 18 leaving each medical college to determine the best ways to meet the standard. 19 This standard (ED-23) has evolved over the years and currently states, “A medical education program must include instruction in medical ethics and human values and require its medical students to exhibit scrupulous ethical principles in caring for patients and in relating to patients' families and to others involved in patient care”. 20 Fewer than 50% of the 141 colleges that belong to the American Association of Medical Colleges (AAMC) actually teach ethics as part of a directed curriculum, 21 so it is likely that the majority of medical colleges incorporate the topic of ethics into other parts of the curricula. Some have suggested that medical education relies on a tacit system and a hidden curriculum, often couched in the teaching of “professionalism”. 22,23 However, this is problematic, since research has shown that courses involving the discussion or practice of ethics have a greater effect on moral judgment than a sublimated moral curriculum, regardless of the nature of the profession. 24,25 The mainstream of research supports a positive relationship between formal education and increased moral judgment, especially if there is any curricular focus on issues of ethics and morality. 24-30 At the time of this study, the curriculum at the research institution included a course titled “Prevention, Community, and Culture” (PCC). Taught in five week-long blocks, spread throughout the first two years of medical school, the course focused on patient care, utilizing cases structured along a preventive medicine theme. It included human behavior issues, ethics, professionalism, alternative and complementary medicine, nutrition, and epidemiology. Clinicians facilitated small-group discussions. An ethicist participated in writing cases and provided at least one lecture on ethical decision-making in medicine; however, there was no training in teaching ethics for the clinician-facilitators, nor was there any evaluation of gains in the students’ ethical perspectives. 

The physician’s moral perspective plays a role in a patient’s quality of life and, indeed, whether that patient lives or dies. It would seem that development of ethical decision-making should be an important component of medical education. Given this, it is surprising that there is not more research using reliable and valid instruments to determine whether medical education is impacting moral reasoning in its students. 16 The few studies that have been conducted report widely varied findings, ranging from no significant differences 24,26 to statistically significant increases in moral reasoning. 27,28 Some studies report declines in moral judgment as a result of medical education. Patenaude, Niyonsenga, and Fafard^29^ longitudinally assessed development in medical students and found significant decreases in their moral judgment. (It should be noted that Jang argued that the interpretation by Patenaude et al. was a misapplication of statistical inference). 30 Lind’s longitudinal study indicated that medical students showed an overall decline of about one percent in their moral judgment competence while other university students in general studies showed an increase of approximately 5.4%. 31 Lind concluded that the decrease was due to the lack of focus on ethics and morality in the medical curriculum. Chalmers et al. 32 and Wiggleton et al. 33 also found decline in the moral reasoning and reflective abilities of students who were in the final stages of medical training. Hren, Marušić, and Marušić theorized that a decline in moral reasoning was correlated to the clinical teaching process, which involved students being told what to do by doctors rather than having the chance to use metacognitive skills to determine a resolution. 34

The purpose of this study was to look at the differences in moral judgment between and among students at the University of Tennessee College of Medicine, part of a large health sciences center located in the south-eastern United States. The research was guided by two questions: 1) Are there significant differences in the moral judgment levels of medical students depending upon their placement in the curriculum, especially between M4 students at the end of their clinical experience and other students? 2) Did exposure of M1 and M2 students to a course focusing on professionalism result in any difference in moral reasoning compared to those who were not exposed to that curriculum (M3 and M4 students)? 

##  Methods

###  Study design and participants

This study was a quantitative, cross-sectional study. The target participants (N=593) were students from all four years of medical school (M1-M4). This was a fairly homogeneous sample, as the majority of the students were Caucasian, and a majority of the respondents were male. All students had at least a baccalaureate degree. The M1 and M2 students had participated in the PCC course, but the M3 and M4 students had not. IRB approval was provided by both the researcher’s institution (the University of Memphis) as well as by the students’ institution (the University of Tennessee Health Science Center). Participants were recruited via email. 

###  Data collection procedures

The Defining Issues Test (DIT) has been widely utilized to measure moral judgment since its introduction in the 1970s, and it has been shown to be a reliable and valid measure of characteristics of moral judgment. 9,35-37 Several studies using the DIT have examined the moral reasoning of student cohorts, including nursing, 38 -40 medicine, 32,41-43 engineering, 44 business, 45-48 teaching, 49veterinary medicine, 50 law, 51 seminary, 52and journalism. 53 Because of its broad use in the research on moral development, this instrument was utilized for this study. 

The DIT consists of six moral dilemmas; the short form of the DIT consists of the first three dilemmas. (One of the most commonly known dilemmas is the “Heinz Dilemma”, which describes a man whose dying wife cannot get needed medication because the local pharmacist is charging too much for the drug, leading Heinz to break into the pharmacy and steal the drug). At the conclusion of each dilemma, participants decide what the outcome of the dilemma should be. Twelve statements are associated with each scenario, and participants rate the importance that each statement had on their decision regarding the outcome. The four highest-ranked items are considered in the scoring of the DIT to determine the relative importance that the participant places on Kohlberg’s post conventional level of moral development. 37 The resulting P Score represents the degree to which the participant utilizes post conventional thinking to solve moral problems. 9

For the purpose of this study, the short form of the DIT, encompassing the first three stories, was utilized. This was because use of the six-item test would likely deter participants from finishing the instrument due to the required time commitment. Participants accessed the DIT via a secure, web-based assessment system in the final month of their respective years of medical school. Data were collected in text format and downloaded into both Microsoft Excel and Microsoft Access. Analysis of the data utilized SPSS. 

###  Data analysis

A total of 192 students responded. Of those, 27 scores were invalidated because of incomplete answers; this left 165 records to be analyzed. Using ANOVA, three scale metrics, the P Score, the Stage Triad, and the Stage, were calculated for each participant.54 The P Score represents the percentage of post conventional thought conveyed by the participant. The Stage Triad is determined by placing the participant’s P Score in one of three levels: lower (0-27%), middle (28-41%), and upper (42-95%). The Stage metric represents the participant’s stage of moral judgment. Cronbach’s Alpha for the three constructs was .752; all analyses utilized an alpha of .05. Except where noted, the test for homogeneity of variance, using the Levene statistic, was not significant, indicating that the assumptions underlying ANOVA were met. Effect sizes were calculated using SPSS and are reported within the ANOVA tables utilizing η^2^. 

##  Results

For all participants (N=165), the mean P Score (the percentage of postconventional thought used) was .42 with a standard deviation of .175. Stage Triad scores (represented by 1 for the lowest third, 2 for the middle third, and 3 for the upper third) show a mean of 2.29 with a standard deviation of 0.83. The mean for Stage score was 4.41, which is in the conventional level of moral judgment; this average had a standard deviation of 0.562.

**Table 1.** Students by graduating class (ANOVA) (n=165)

Source		df	F	η^2^	p
P Score	Between Groups	3	.356	.145	.758
	Within Groups	161			
	Total	164			
Stage Triad	Between Groups	3	.102	.001	.959
	Within Groups	161			
	Total	164			
Stage	Between Groups	3	.201	.028	.896
	Within Groups	161			
	Total	164			

##  Differences in students depending upon placement in the curriculum

The Levene Statistic (F_(3, 161)_ = 2.696, p < .05) was significant on the Stage variable, so an alpha of .10 was utilized. When comparing the M1, M2, M3, and M4 classes, no significant differences were found between the student cohorts when testing for P Score (F_(3, 161)_ = .356, p < .10, Effect Size= .145), Stage (F_(3, 161)_ = .201, p < .10, Effect Size= .028), or Stage Triad (F_(3, 161)_ = .102, p < .10, Effect Size = .001). In each case, the p was greater than the utilized alpha of .10. These results are reported in Table 1.

**Table 2:** Students who had completed curriculum vs. those who had not (n=165)

Source	Class	n	M	SD
P Score	M1-M3	135	.415	.174
	M4	30	.451	.179
	Total	165	.422	.175
Stage Triad	M1-M3	135	2.28	.843
	M4	30	2.30	.794
	Total	165	2.28	.832
Stage	M1-M3	135	4.39	.574
	M4	30	4.47	.507
	Total	165	4.41	.562

The educational experiences of graduating M4 students are vastly different from those students still involved in the M1,M2, and M3 years, so the data was analyzed to look for any changes due to those experiences. The test for homogeneity of variance was not significant for P Score (F_(1, 163)_ = .779, p > .05), Stage Triad (F_(1, 163)_ = .900, p > .05), or Stage (F_(1, 163)_= . 712, p > .05), indicating that the assumptions underlying the application of ANOVA were met. As Table 2 and Table 3 illustrate, ANOVA showed no significant differences between the M4 students when compared to the M1, M2, or M3 students in the P Score (F_(1, 163)_ = 1.029, p > .05, Effect Size = .10), Stage Triad (F_(1, 163)_ = 0.012, p > .05, Effect Size = .01), or Stage (F_(1, 163)_ = 0.425, p > .05, Effect Size = .07).

##  Differences from exposure to professionalism course

Were there any differences between those students who had experienced a slightly more focus in ethics teaching (M1 and M2 students) compared to those who had not (M3 and M4 students)? Homogeneity of variance was not sufficient for Stage (F_(1, 163)_ = 6.895, p < .01), so unequal variances were assumed. This analysis showed no significant differences in P Score (t_163_ = -0.553, p>.05), Stage Triad (t_163_=0.015, p>.05), or Stage (t_162.98 _= 0.0138, p=.01) between the two cohorts making up the M1/M2 students and the M3/M4 students (Table 4  and Table 5).

**Table 3:** Students who had completed curriculum vs. those who had not (ANOVA) (n=165)

Source		df	F	η^2^	p
P Score	Between Groups	1	1.029	.10	0.312
	Within Groups	163			
	Total	164			
Stage Triad	Between Groups	1	0.0121	.01	0.913
	Within Groups	163			
	Total	164			
Stage	Between Groups	1	0.425	.07	0.515
	Within Groups	163			
	Total	164			

##  Discussion

The purpose of this study was to compare the moral development of medical students at various stages of their medical education. The study found no significant differences in moral judgment between medical students in any of the separate curricular years. Data analysis showed no differences between those who had participated in a course of study that included some emphasis on ethics, nor did it show differentiation between those students who had been involved in hands-on clinical experience compared to those students who had not. Thus, there seemed to be no significant progress in moral reasoning taking place during the four years of medical education. Much more importantly, most students did not exhibit postconventional thinking and operated within the conventional level of moral processing. This reflects a tendency to think within a set of rules, instead of focusing on what may be best for the patient. Rules, in the context of medical practice, can include local practice standards; national, evidence-based standards of care; and cost limits established by clinics and hospitals. 55

**Table 4:** M1 and M2 students vs. M3 and M4 students (n=165)

Source	Cohort	n	M	SD
P Score	M1/M2	91	0.415	0.174
	M3/M4	74	0.430	0.177
	Total	165	0.422	0.175
Stage Triad	M1/M2	91	2.286	0.847
	M3/M4	74	2.284	0.820
	Total	165	2.285	0.832
Stage	M1/M2	91	4.407	0.614
	M3/M4	74	4.405	0.494
	Total	165	4.406	0.562

**Table 5:** M1 and M2 students vs. M3 and M4 students t-test (n=165)

Source	* *	Levene's test for equality of variances		t-test for equality of means						
		F	p	t	df	p (2-tailed)	Mean difference	Standard error difference	95%CI	
									Lower	Upper
P Score	Equal variances assumed	0.344	0.559	-0.553	163	0.581	-0.015	0.027	-0.069	0.039
	Equal variances not assumed			-0.551	154.88	0.582	-0.015	0.028	-0.070	0.039
Stage	Equal variances assumed	6.895	0.010	0.0135	163	0.989	0.001	0.088	-0.173	0.175
	Equal variances not assumed			0.0138	162.98	0.989	0.001	0.0863	-0.169	0.172
Stage Triad	Equal variances assumed	0.382	0.537	0.015	163	0.988	0.002	0.131	-0.256	0.260
	Equal variances not assumed			0.015	158.12	0.988	0.002	0.130	-0.255	0.259

In this study, the average P score (42%) of these future doctors was more similar to that of the “average college student” (42.3%) than to the reported average of practicing medical physicians (49.5%).^55^ One might argue that these students were not long out of college, but age alone has not been shown to be related to DIT scores in adult groups, 54 so this single variable would not explain the gap between the medical students and the physicians. One possible explanation for the difference in scores could be the real experience that occurs in the years beyond medical school, including residency training and professional practice, when the medical doctor has much more responsibility for patient care. It would make sense that a physician’s moral reasoning would evolve from the actual practice of medicine. 56

The four years of medical education generally provide extremely diverse experiences for most medical students. From the didactic, lecture-heavy basic science (M1 and M2) years to the hands-on, situational learning opportunities of the clinical (M3 and M4) years, students in this study experienced a wide range of information, both in content and in presentation, and a wide range of learning experiences, from passive lecture to active learning in the clinic. During the first two years of medical school, with limited exposure to the practice of medicine or to patients, little in the curriculum challenges any student’s moral status quo. For these students, the first two years of medical education generally provided few metacognitive activities and little occasion for the kind of thinking that could influence moral development. It is likely that those developing the PCC curriculum hoped that discussions about professionalism would encourage some increase in moral perspectives, but the moral judgments of students who had participated in the PCC course were no different than those of students who had not. This is not entirely surprising. Rhodes and Cohen note that the “nature and content of these fields [medical ethics and professionalism] and their relationship to one another remains confused and vague”. 57

During the last two years of medical education, students rotated through different clinical settings and participated with supervising physicians in making decisions with significant impact on the patient and the patient’s family. Kohlberg theorized that interaction with the environment, in concert with cognitive prerequisites, would provide the catalyst for change in the individual’s perspective of the self and the other, impacting moral perspective. It is interesting, then, that this study showed no differences in moral reasoning by students who had experienced both the knowledge acquisition and the hands-on clinical experiences. Even the M4 students, in their last month of medical school and having had the most exposure to patient care, showed no significant differences in moral reasoning when compared to the other students. 

Encounters with situations concerning patient well-being, disease treatment, and end-of-life issues would likely trigger the kind of experiences that Kohlberg thought necessary for progression in moral reasoning. However, students in clinical training work under the supervision of attending physicians and residents and are, therefore, at the bottom of the clinical chain of command. They are also subject to the powerful tacit learning system that is present in clinics and hospitals. 58 Branch theorized that the lack of moral development of medical students relates to perceived pressures to conform to the culture of the medical wards. 59 Similarly, Wiggleton et al. and Hren et al. theorized moral distress as a result of the hierarchy found in the clinical setting, rather than from the challenges experienced in patient care. 33, 34 Whatever the cause, the fact that medical students are processing decisions in the conventional, rather than the postconventional level, indicates a significant problem and should be cause for concern. 

This study had several limitations. The most significant is the homogeneity of the participants. The majority of the participants were male and Caucasian, restricting the generalizability of the findings. Another limitation is the number of participants; 32.3% of the student body participated, which represents a very small percentage of medical students worldwide. Finally, a longitudinal study would provide a richer source of data to determine what, if any, impact the medical school curriculum has on the moral development of its students, helping to determine how subsequent experience accounts for changes in moral judgment.

##  Conclusions

Hren et al. noted that “current evidence for the relationship between education and moral reasoning does not clearly apply to medical students”, 34 The current curriculum for developing moral reasoning is not effective, and a more directed program of ethics education needs to be implemented in medical education. This approach has empirical support. 17, 27, 28, 60 As medical technology advances, medical interventions increasingly influence the physician’s decision-making. However, not all technological advances improve the patient’s quality of life or even extend the patient’s lifespan to a significant degree. Medical staff must consider solutions that consider the values held by the patients. If students entering medical schools do not function at a postconventional level, allowing them to think past their own benefit or the “rules” of medical intervention, it is the responsibility of medical educators to push students to develop more complex ethical thinking. Medical education must re-focus its efforts to produce healthcare professionals who are not only competent with their skills but are also sufficient in their moral reasoning abilities. A growing focus on medical and bioethics challenges medical educators to present their students with consistent, pertinent, and focused educational opportunities meant to improve moral reasoning and metacognitive skills. Our lives - and the quality of our lives - may depend on it. 

## References

[1]  Hinkka  H,  Kellokumpu-Lehtinen  P,  Kosunen  E,  Lammi  UK,  Metsanoja  R (2002). Factors affecting physicians' decisions to forgo life-sustaining treatments in terminal care.. J Med Ethics.

[2]  Christakis   NA ,  Asch   DA  (1995). Physician characteristics associated with decisions to withdraw life support.. Am J Public Health.

[3]  Cohen  C,  Chen  Y,  Orbach  H,  Freier-Dror  Y,  Auslander  G,  Breuer  G (2014). Social values as an independent factor affecting end of life medical decision making.. Med Health Care Philos.

[4]  Derryberry  WP,  Thoma  SJ (2005). Moral judgment, self-understanding, and moral actions: the role of multiple constructs.. Merrill-Palmer Quarterly.

[5]  Thoma  SJ,  Rest   JR,  Narváez  D (1994). Moral judgments and moral action.. Moral development in the professions psychology and applied ethics.

[6]  Piaget  J (1969). .. The child's conception of the world..

[7]  Kohlberg  L (1958). The development of modes of moral thinking and choice in the years 10 to 16.

[8]  Rest   JR ,  Narváez   D  (1994). Moral development in the professions: psychology and applied ethics..

[9]  Rest   JR  (1979). Development in judging moral issues..

[10]  Piaget   J  (1965). The moral judgment of the child..

[11]  Kohlberg   L  (1971). Moral judgment interview and procedures for scoring..

[12]  Turiel   E  (1983). The development of social knowledge: morality and convention..

[13]  Bandura   A  (2002). Selective moral disengagement in the exercise of moral agency..

[14]  Kohlberg   L  (1981). The philosophy of moral development: moral stages and the idea of justice..

[15]  Power   F ,  Clark   H ,  D'Alessandro   A ,  Kohlberg   L  (1989). Lawrence Kohlberg's approach to moral education..

[16]  Self   DJ,  Baldwin   DC Jr ,  Rest   JR ,  Narváez  D (1994). Moral reasoning in medicine.. Moral development in the professions..

[17]  Lakhan   SE ,  Hamlat   E ,  McNamee   T ,  Laird   C  (2009). Time for a unified approach to medical ethics..

[18]  Doukas   DJ ,  McCullough   LB ,  Wear   S  (2012). Perspective: medical education in medical ethics and humanities as the foundation for developing medical professionalism.. Acad Med.

[19]  Bebeau   MJ ,  Monson   VE ,  Nucci   LP ,  Narváez  D (2008). Guided by theory, grounded in evidence: a way forward for professional ethics education.. Handbook of moral and character education..

[20] Liaison Committee on Medical Education Functions and structures of a medical school. Standards for accreditation of medical education programs leading to the MD degree.. http://www.lcme.org/publications/functions.pdf.

[21]  Peppler   R  Message from lnme-314a bizhub c452. [Internet]. Message to: Murrell VS. 2013 July 27.

[22]  Huddle  TS (2005). Teaching professionalism: is medical morality a competency?. Acad Med.

[23]  Stern  DT,  Papadakis  M (2006). The developing physician--becoming a professional.. N Engl J Med.

[24]  Bebeau   MJ  (2002). The Defining Issues Test and the four component model: contributions to professional education.. J Moral Educ.

[25]  Schlaefli  A (1985). Does moral education improve moral judgment? A meta-analysis of intervention studies using the Defining Issues Test.. Rev Educ Res.

[26]  Self  DJ,  Baldwin   DC Jr  (1998). Does medical education inhibit the development of moral reasoning in medical students? A cross-sectional study.. Acad Med.

[27]  Self  DJ,  Baldwin   DC Jr ,  Wolinsky   FD  (1992). Evaluation of teaching medical ethics by an assessment of moral reasoning.. Med Educ.

[28]  Self  DJ,  Olivarez  M,  Baldwin   DC Jr  (1998). Clarifying the relationship of medical education and moral development.. Acad Med.

[29]  Patenaude  J,  Niyonsenga  T,  Fafard  D (2003). Changes in the components of moral reasoning during students' medical education: a pilot study.. Med Educ.

[30]  Jang  RW (2003). Does medical education blunt our moral reasoning? A different interpretation of the Sherbrooke moral development study.. Univ Toronto Med J.

[31]  Lind  G (2000). Moral regression in medical students their learning environment.. Rev Bras Educ Med.

[32]  Chalmers  P,  Dunngalvin  A,  Shorten  G (2011). Reflective ability and moral reasoning in final year medical students: a semi-qualitative cohort study.. Med Teach.

[33]  Wiggleton  C,  Petrusa  E,  Loomis  K,  Tarpley  J,  Tarpley  M,  O'Gorman  ML (2010). Medical students' experiences of moral distress: development of a web-based survey.. Acad Med.

[34]  Hren  D,  Marušić  M,  Marušić  A (2011). Regression of moral reasoning during medical education: combined design study to evaluate the effect of clinical study years.. PLoS One.

[35]  Rest  JR,  Narváez  D,  Bebeau  MJ,  Thoma  SJ (1999). Postconventional moral thinking: a neo-Kohlbergian approach..

[36]  Thoma   SJ ,  Dong   Y  (2014). The Defining Issues Test of moral judgment development.. Behavioral Development Bulletin.

[37]  White   RD (1999). Are women more ethical? Recent findings on the effects of gender upon moral development.. Journal of Public Administration Research and Theory.

[38]  Duckett   L ,  Rowan   M ,  Ryden   M,  Krichbaum   K ,  Miller   M ,  Wainwright   H  (1997). Progress in the moral reasoning of baccalaureate nursing students between program entry and exit.. Nurs Res.

[39]  Comrie   RW  (2012). An analysis of undergraduate and graduate student nurses’ moral sensitivity.. Nurs Ethics.

[40]  Park   M,  Kjervik   D ,  Crandell   J ,  Oermann   MH  (2012). The relationship of ethics education to moral sensitivity and moral reasoning skills of nursing students.. Nurs Ethics.

[41]  Kumagai   AK  (2008). A conceptual framework for the use of illness narratives in medical education.. Acad Med.

[42]  Slováčková   B ,  Slováček   L  (2007). Moral judgement competence and moral attitudes of medical students.. Nurs Ethics.

[43]  Morton   KR ,  Worthley   JS ,  Testerman   JK ,  Mahoney   ML  (2006). Defining features of moral sensitivity and moral motivation: pathways to moral reasoning in medical students.. J Moral Educ.

[44]  Magun-Jackson   S  (2004). A psychological model that integrates ethics in engineering education.. Sci Eng Ethics.

[45]  Bay   DD ,  Greenberg   RR  (2001). The relationship of the DIT and behavior: a replication.. Issues in Accounting Education.

[46]  Schmidt   CD ,  McAdams   CR ,  Foster   V  (2009). Promoting the moral reasoning of undergraduate business students through a deliberate psychological education‐based classroom intervention.. J Moral Educ.

[47]  Weeks   WA ,  Moore   CW ,  McKinney   JA ,  Longenecker   JG  (1999). The effects of gender and career stage on ethical judgment.. J Bus Ethics.

[48]  Shepard   JM ,  Hartenian   LS  (1991). Egoistic and ethical orientations of university students toward work-related decisions.. J Bus Ethics.

[49]  Chang   F-Y ,  Rest  JR,  Narváez  D (1994). School teachers' moral reasoning.. Moral development in the professions: psychology and applied ethics..

[50]  Self  DJ,  Olivarez   M ,  Baldwin   DC Jr ,  Rest   JR ,  Narváez   D  (1994). Moral reasoning in veterinary medicine.. Moral development in the professions: psychology and applied ethics.

[51]  Barry   BE ,  Ohland   MW  (2009). Applied ethics in the engineering, health, business, and law professions: a comparison.. Journal of Engineering Education.

[52]  Bunch   WH  (2005). Changing moral judgement in divinity students.. Journal of Moral Education.

[53]  Westbrook   T ,  Rest   JR ,  Narváez  D (1994). Tracking the moral development of journalists: a look at them.. Moral development in the professions: psychology and applied ethics..

[54]  Rest   JR  (1990). DIT manual: manual for the Defining Issues Test.

[55]  Lewis   M ,  Gohagan   JK ,  Merenstein   DJ  (2007). The locality rule and the physician's dilemma: local medical practices vs the national standard of care.. JAMA.

[56]  Narváez   D ,  Bock   T  (2002). Moral schemas and tacit judgement or how the Defining Issues Test is supported by cognitive science.. Journal of Moral Education.

[57]  Rhodes   R ,  Cohen   DS  (2003). Understanding, being, and doing: medical ethics in medical education.. Camb Q Healthc Ethics.

[58]  Coulehan   J ,  Williams   PC  (2003). Conflicting professional values in medical education.. Camb Q Healthc Ethics.

[59]  Branch   WT Jr  (2000). Supporting the moral development of medical students.. J Gen Intern Med.

[60]  Self   DJ ,  Wolinsky   FD ,  Baldwin   DC Jr  (1989). The effect of teaching medical ethics on medical students' moral reasoning.. Acad Med.

